# Pregnancy after mumps: a case report

**DOI:** 10.1186/s13256-019-2271-9

**Published:** 2019-12-22

**Authors:** Parviz Shahabi, Shiva Asadzadeh, Hossein Bannazadeh Baghi, Behnaz Sadeghzadeh Oskouei

**Affiliations:** 1 Drug Applied Research Center, Tabriz University of Medical Sciences, Tabriz, Iran; 2 Immunology Research Center, Tabriz University of Medical Sciences, Tabriz, Iran; 3 Infectious and Tropical Diseases Research Center, Tabriz University of Medical Sciences, Tabriz, Iran; 4 Department of Midwifery, Nursing and Midwifery Faculty, Tabriz University of Medical Sciences, Tabriz, Iran; 5 Reproductive Biology, Tabriz University of Medical Sciences, PO Box 5138947977, Tabriz, Iran

**Keywords:** Mumps, Oophoritis, Premature ovarian failure, Drospil

## Abstract

**Introduction:**

Oophoritis, a complication of mumps, is said to affect only 5% of all postpubertal women. In this report, we present a case of a 31-year-old Iranian woman with amenorrhea and infertility due to an infantile uterus and atrophic ovaries associated with contracting mumps at a young age. She later successfully carried a healthy baby to term.

**Case presentation:**

The patient was diagnosed with oophoritis when she was 8 years of age. She had no menses before treatment. The patient underwent a low-dose contraceptive treatment from age 19 until she was 31 years of age. During this period, the size of her uterus was constantly monitored, which revealed constant yet slow uterine growth. At age 31, Drospil (containing 3 mg of drospirenone and 0.03 mg ethinyl estradiol) treatment was initiated and administered for 3 months, which led to substantial uterine growth and menses. After her uterus had reached a mature size, the patient was referred to an assisted reproductive technology clinic. There she received a donor oocyte that was fertilized with the sperm of her husband. She had a successful low-risk pregnancy after the second embryo transfer.

**Conclusion:**

Low-dose contraceptive treatment containing progesterone, followed by Drospil, which includes both estradiol and progesterone, had a synergistic effect that led to the growth of the patient’s uterus.

## Introduction

Mumps is an acute contagious viral infection that occurs mainly in school-aged children or adolescents and is caused by a single-stranded ribonucleic acid (RNA) virus. Clinical manifestations of the infection most often include orchitis, oophoritis, aseptic meningitis, encephalitis, deafness, and pancreatitis. However, most worrying is that at least one of these complications occurs in 47% of patients infected with mumps [1]. Although some complications of the disease are well researched, there are others that scientists know very little about. Primary examples are in the clinical manifestation of orchitis and oophoritis. Both of these complications of mumps are postpubertal complications that affect the reproductive area of men and women, respectively. Although orchitis is established as the most common complication of mumps in postpubertal men (20–30% of all cases), oophoritis is said to affect only 5% of all postpubertal women. However, long-term studies cast doubt on these findings because the acute symptoms of oophoritis are not always linked to the disease [2]. In contrast to orchitis, the occurrence of oophoritis is easily overlooked because of the anatomical position of the ovaries. Also, oophoritis may often mimic the acute abdominal pain of appendicitis, and most effects of the infection are experienced on a long-term basis [3].

Often oligomenorrhea or even amenorrhea, which are common effects of oophoritis, might be attributed to other causes and could lead affected women to seek out ineffective fertility and hormone treatments. However, the absence or underdevelopment of the uterus can be a sign of oophoritis. These factors have led especially to developing countries taking a closer look at the link between infertility and oophoritis and further research into possible oophoritis treatments.

## Case presentation

Our patient was a 31-year-old Iranian woman with amenorrhea and infertility due to an infantile uterus and atrophic ovaries associated with contracting mumps at a young age. She ultimately successfully carried a baby to term after a uterine growth treatment. The patient had contracted mumps when she was 3 years old and was diagnosed with oophoritis when she was 8 years of age. She had had no menses before treatment and had a body mass index (the weight in kilograms divided by the square of the height in meters) of 23.3. Her initial reason for undergoing treatment was that she wished to conceive a child and carry it to term.

### Clinical findings

The patient received a donor oocyte that was fertilized with the sperm of her husband. She had a successful low-risk pregnancy after the second embryo transfer.

### Timeline

**Fig. 1:**
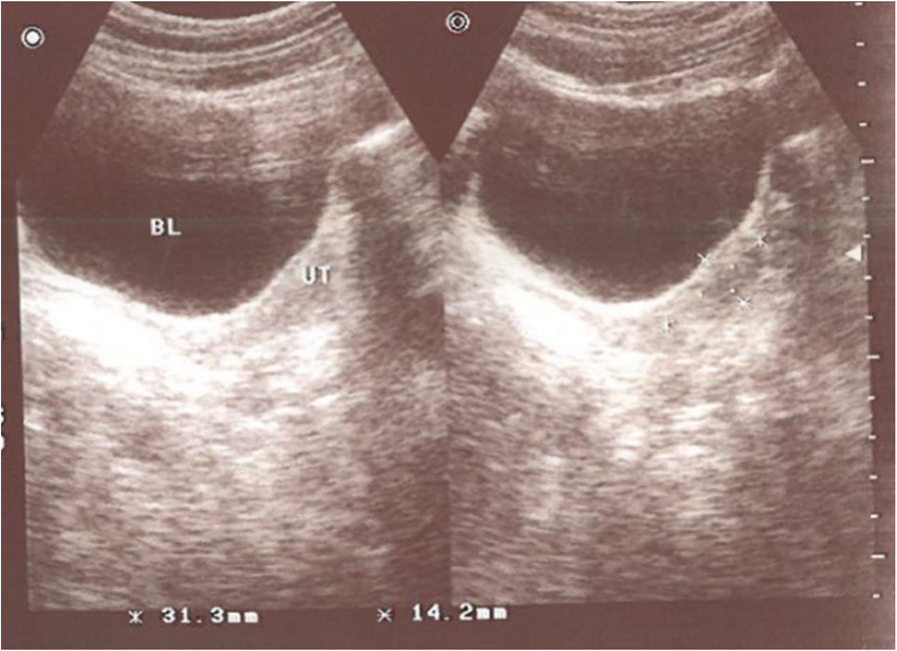
Ultrasound of patient’s uterus at 19 years of age. Uterus measures 31 × 14 mm

**Fig. 2:**
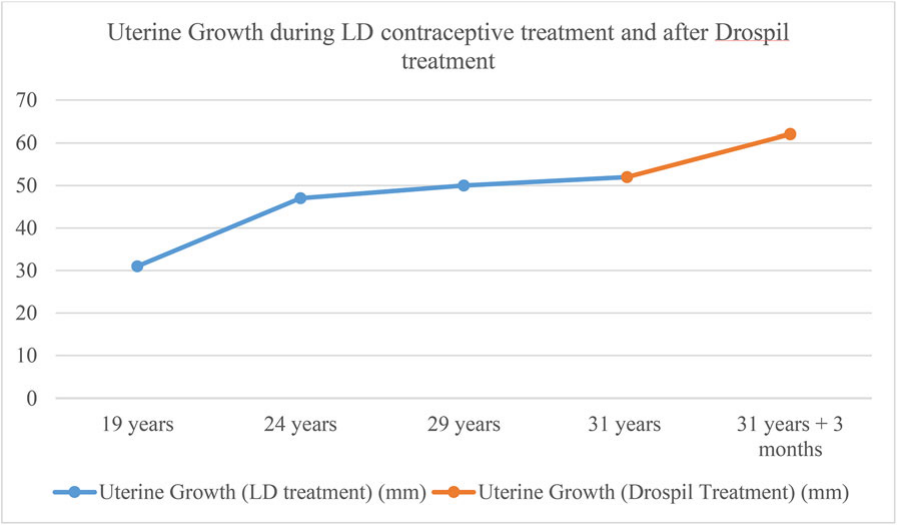
Timeline of uterine growth during low-dose contraceptive treatment and after Drospil treatment. The timeline does not increase in equal time increments but simply illustrates uterine growth during important stages of the infertility treatment. The *y*-axis indicates millimeters. The *blue line* shows the uterine growth in millimeters during the low-dose contraceptive treatment that spans over 12 years. The *orange line* shows the uterine growth in millimeters during the Drospil treatment, which lasted for 3 months

**Fig. 3:**
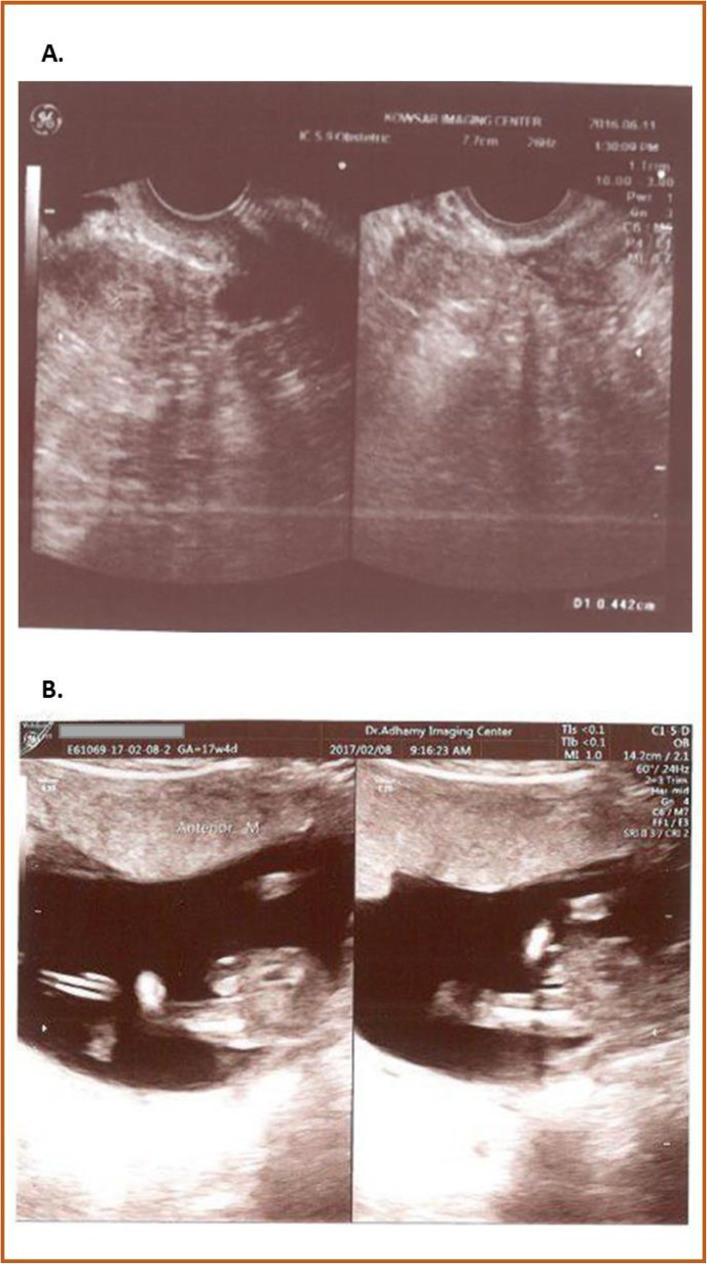
**a** Ultrasound of patient’s uterus at 31 years of age after Drospil treatment. Uterus measures 65 × 28 mm. **b** Pregnancy ultrasound at 17 + 4 weeks

During her low-dose (LD) contraceptive treatment, the patient experienced slow but steady uterine growth. Her uterus measured 31 × 14 mm when she was 19 years old (Fig. 1), which was 4 years into her LD contraceptive treatment. At 24 and 29 years of age, her uterus measured 47 × 13 mm and 50 × 17 mm, respectively. At the end of the LD contraceptive treatment, when she was 31 years old, her uterus had grown to 52 × 15 mm. Drospil (oral contraceptive tablets containing 3 mg of drospirenone and 0.03 mg of ethinyl estradiol; Koushan Pharmed, Iran, Tehran) was administered when the patient was 31 years of age, and this treatment lasted 3 months. During this time, her uterus grew a substantial amount (Fig. 2). The subject’s uterus grew from 52 × 15 mm to 65 × 28 mm (Fig. 3) after the Drospil treatment.

### Diagnostic assessment

The patient underwent LD contraceptive treatment from age 19 until she was 31 years of age. During this period, the size of her uterus was constantly monitored, which revealed constant yet slow uterine growth. This LD contraceptive treatment is routine and serves as a hormone replacement therapy to prevent premature menopause side effects and is a standard agent for menstrual period creation. At age 31, Drospil treatment was initiated and administered for 3 months, which led to substantial uterine growth and menses. After her uterus had reached a mature size, the patient was referred to an assisted reproductive technology clinic.

After her LD contraceptive treatment, the patient experienced only a small amount of withdrawal bleeding. During the Drospil treatment, the patient started experiencing menses with massive withdrawal bleeding. Additionally, the endometrial lining, at the 14 days menstrual cycle day, thickened from 2 mm before the Drospil treatment to 6 mm after the Drospil treatment.

### Follow-up and outcomes

The Drospil treatment lasted a total of 3 months, after which the patient’s uterus size and function were deemed to be mature. After this treatment, a healthy low-risk pregnancy (Fig. 3) was carried to term.

## Discussion

Currently, the mean age of menopause is approximately 51 years [4]; however, pathogenic conditions that cause premature ovarian failure (POF), which are determined by elevated follicle-stimulating hormone levels above 40 IU/L, hinder many women from successfully conceiving children [5]. This is because POF causes symptoms such as amenorrhea due to the cessation of ovarian function before the age of 40, elevated gonadotropins, and infertility [6]. Causes of POF include genetic, autoimmune, metabolic, iatrogenic, idiopathic, and infectious, as was the case in our case patient [3]. We report a successful fertility treatment of a 31-year-old woman with POF that was due to contracting the infectious disease mumps. We were able to successfully grow her uterus through LD contraceptive treatment followed by a more intensive 3-month Drospil treatment. It is believed that the LD contraceptive treatment, which contained progesterone, followed by Drospil, which includes both estradiol and progesterone, had a synergistic effect that led to the growth of the patient’s uterus. It has been proved that estrogen and progesterone have uterotrophic potential because of their antagonistic and synergistic effects. The growth-promoting effects are both hypertrophic and hyperplastic. Recent studies show that treatment with progesterone before estradiol leads to hypertrophic and hyperplastic growth of the uterus. When progesterone is administered after estradiol, a suppressive effect on growth is experienced. However, progesterone administration before estradiol has a synergistic effect on DNA synthesis. This is why we believe that administering Drospil after a routine LD contraceptive treatment can be of benefit to grow the size of the uterus of POF candidates.

## Conclusion

The LD contraceptive treatment, which contained progesterone, followed by Drospil, which includes both estradiol and progesterone, had a synergistic effect that led to the growth of the patient’s uterus.
